# Reimagining Mutual Aid to Advance Healthy Outcomes Among Older Grandparent Caregivers

**DOI:** 10.1093/geroni/igaf122.1173

**Published:** 2025-12-31

**Authors:** Tina Peterson

**Affiliations:** University of Cincinnati, Cincinnati, Ohio, United States

## Abstract

African American grandparents raising grandchildren vary in their use of informal and formal services. These differences in service utilization can emerge from societal influences that limit access to needed services and resources. African American grandparents raising grandchildren may compensate by developing mutual aid support networks. Yet, little is known about mutual aid observed among African American grandparents raising grandchildren. This research reports on mutual aid described by older African American grandparents raising grandchildren. The sample included eleven African American grandparent caregivers who participated in two separate qualitative research studies between 2015 and 2024. All grandmothers were primary caregiver for a grandchild and 40 years or older. In-depth interviews were audiotaped, transcribed verbatim, and analyzed for themes. A common theme among these African American grandmothers was the intersection between caregiving, hypertension, and stress. Mutual aid evolved from historical experiences with informal and formal services. African American grandmothers linked caregiving processes to past role models of caregiving within their family of origin. Mutual aid also consisted of exchanges with family, friends, coworkers, neighbors, and peers. Multiple forms of mutual aid manifested as information sharing to encourage and prevent health-related crises, sharing financial resources, coping with the demands of raising grandchildren, volunteering, identifying healthier recipes and meal exchanges, and distributing vegetable from personal gardens. More emphasis is needed to identify the distinct sources and contributions of mutual aid systems. Mutual aid can help to advance healthy outcomes when resource and service gaps exist in informal and formal social support networks.

